# Influence of High, Disperse API Load on Properties along the Fused-Layer Modeling Process Chain of Solid Dosage Forms

**DOI:** 10.3390/pharmaceutics11040194

**Published:** 2019-04-22

**Authors:** Marius Tidau, Arno Kwade, Jan Henrik Finke

**Affiliations:** 1 Institute for Particle Technology, TU Braunschweig, Volkmaroder Str. 5, 38104 Braunschweig, Germany; a.kwade@tu-braunschweig.de (A.K.); jfinke@tu-braunschweig.de (J.H.F.); 2 Center of Pharmaceutical Engineering, TU Braunschweig, Franz-Liszt-Str. 35A, 38106 Braunschweig, Germany

**Keywords:** additive manufacturing, modified release, filament extrusion, fused layer modeling, theophylline, high API load

## Abstract

In order to cope with the increasing number of multimorbid patients due to demographic changes, individualized polypill solutions must be developed. One promising tool is fused layer modeling (FLM) of dosage forms with patient-specific dose combinations and release individualization. As there are few approaches reported that systematically investigate the influence of high disperse active pharmaceutical ingredient (API) loads in filaments needed for FLM, this was the focus for the present study. Different filaments based on polyethylene oxide and hypromellose (HPMC) with different loads of theophylline as model API (up to 50 wt.%) were extruded with a twin-screw extruder and printed to dosage forms. Along the process chain, the following parameters were investigated: particle size and shape of theophylline; mechanical properties, microstructure, mass and content uniformity of filaments as well as dosage forms and the theophylline release from selected dosage forms. Especially for HPMC, increasing theophylline load enhanced the flexural strength of filaments whilst the FLM accuracy decreased inducing defects in microstructure. Theophylline load had no significant effect on the dissolution profile of HPMC-based dosage forms. Therefore, a thorough analysis of particle-induced effects is necessary to correlate mechanical properties of filaments, printability, and the dosage-and-release profile adjustment.

## 1. Introduction

Considering the demographic changes of society and the connected increase in multimorbid patients, new individualized therapies must be developed including the combination of highly dosed formulations in a single dosage form to improve patient compliance. To produce dosage forms with individual doses and release profiles, new production processes are necessary. As a promising tool for the production of individualized solid dosage forms, additive manufacturing techniques have emerged in pharmaceutical research in the past two decades [1,2]. With these techniques, three-dimensional objects are made without molds or tools by successive build-up of layers of source materials [3]. Depending on the source material, different physical principals are applicable, like powder-bed binding via laser sintering or binder fluid, extrusion freeforming, and stereo lithography [3]. All named techniques are also applicable for the production of solid dosage forms [2]. The first and only approved additively manufactured pharmaceutical. Spritam^®^, is produced by the ZipDose^®^ technology where a powder bed of levetiracetam is selectively bound with a binder fluid to generate an ultra-rapidly disintegrating tablet [4]. However, as there are only few different doses approved and only one dissolution profile is available, Spritam^®^ does not utilize all advantages of additive manufacturing to produce individual dosage forms on demand. Another technique, selective laser sintering, was used to produce orally disintegrating [5] or modified-release tablets [6]. Extrusion-based techniques include the selective deposition of hydrogels loaded with active pharmaceutical ingredients (API) [7,8] which after drying, were tested with up to five different drugs with each having a different excipient to obtain different release profiles [9]. Furthermore, fused layer modeling (FLM), where a polymer-API formulation is selectively deposited via a hot nozzle, also represents extrusion-based technologies and is currently the fastest evolving technique in pharmaceutical research [1].

Few approaches use direct melt extrusion of polymer-API powder blends [10,11]. However, most FLM machines use polymer wires, so-called filaments, as source material [1]. The first applications of FLM in pharmaceutics soaked commercially available polyvinyl alcohol filaments in API solutions to additively produce dosage forms [12,13]. More recent works use mostly hot melt extrusion (HME) to produce API-loaded filaments [1]. In general, HME of polymer-API blends is an advantageous process to improve solubility of BCS type-II and -IV APIs by intensively compounding the materials to gain solid dispersions and solutions [14]. For more soluble APIs, the compounding in polymeric excipients can be used to achieve modified and control release kinetics. To gain lower viscosities and therefore lower process temperatures in HME, plasticizers are frequently used [15]. However, it has to be considered that every substance in the process can have a plasticizing, viscosity decreasing, or even viscosity increasing effect. For example, absorbed water may plasticize polymers and alter the melting process [16], but can also cause polymer degradation [17] and physical instability of preliminary stable solid solutions [18]. Amorphous, molecularly dispersed APIs also have a plasticizing effect [19,20]. On the contrary, dispersely mixed APIs increase viscosity [21,22]. To produce filaments suitable for FLM machines adequate process parameters for HME and formulation are crucial. Thus, adequate mechanical stability, a consistent diameter, and a homogeneous API distribution are required [23]. There are different excipients of pharmaceutical grade that can be used as matrix polymer or plasticizer [24]. Depending on the excipient used, different release kinetics can be adjusted. It is also possible to mix different polymeric excipients to gain better applicability to FLM [24,25].

For the production of API-loaded filaments, different machinery was applied. For example, filaments were produced by the extrusion of powder blends with a single-screw extruder [23,26] with marginal shear and melt mixing or with a self-constructed ram-extruder [27] without any melt mixing. However, in most cases a co-rotating twin-screw extruder is applied [28,29,30,31] to obtain well-compounded, homogeneous filaments.

Different API-loaded filaments were, as mentioned before, successfully used in FLM to produce immediate [27] and controlled [23,25,28] release solid dosage forms. This includes first approaches with two formulations using a dual FLM machine to obtain delayed release and dosage forms with API combinations [32,33,34]. However, in most cases the used formulations include low API loads [23,33]; only few use API loads higher than 40 wt.% [31,35].

Especially for polypill applications, it is necessary to use source materials with high API loads to limit the dosage form size whilst increasing the maximum dose for each API. There are only few approaches to systematically investigate the influence of highly disperse API load on filament and additively produced dosage form properties. Therefore, the aim of the present work is to investigate the introduction of theophylline as model drug at different concentrations into different polymer matrices via HME. The obtained filaments and additively produced dosage forms were methodically characterized concerning parameters crucial over the whole process chain such as mechanical properties, content and mass uniformity, and drug release.

## 2. Materials and Methods

### 2.1. Materials

Hypromellose (HPMC) suited for HME (AFFINISOL^TM^ HPMC HME 15lv), polyethylene glycole (PEG) with a molecular weight of 8000 g/mol (CARBOWAX^TM^ PEG 8000), semi-crystalline polyethylene oxide (PEO) with a molecular weight of 100,000 (Sentry POLYOX™ WSR N-10–NF Grade;) and one (PEO-L) with a molecular weight of 2,000,000 (Sentry POLYOX™ WSR N-60K–NF Grade) were used as matrix polymers and were kindly donated by Dow (Bomlitz, Germany). As model API, theophylline (≥99%, anhydrous powder) was obtained from Sigma-Aldrich (Darmstadt, Germany). All solvents used were of analytical grade.

### 2.2. Methods

#### 2.2.1. Analysis of Particle Size Distributions

The particle size distributions of raw material powders were measured using laser diffraction (Mastersizer 3000; Malvern Panalytical GmbH, Kassel, Germany) through a dry powder dispersing cell. The pressure of the dispersing air jet was set to 4 bar and the obtained data were evaluated applying Fraunhofer theory. For each material, the average of three measurements over 5 s was calculated and the volumetric distribution with the corresponding 10%, 50% and 90% cumulative undersize (d_10_, d_50_, d_90_) was determined.

#### 2.2.2. Preparation of Theophylline-Loaded Filaments

Filaments with a diameter of 2.85 mm ± 0.1 mm were produced applying a co-rotating twin-screw extruder (KETSE 12/36; Brabender^®^ GmbH & Co. KG, Duisburg, Germany) with a die diameter of 2.7 mm (except for pure HPMC filament that was produced by applying a Pharma 11 HME (ThermoFischer ScientificTM, Karlsruhe, Germany) with a die diameter of 3 mm, obtained filament diameter was also 2.85 mm ± 0.1 mm through pull-off speed adjustment) in rotation speed-controlled mode at 100 rpm at temperatures adapted to formulations (Table 1). Pre-mixed powder blends (Table 1) were fed with a twin-screw gravimetric feeder (Brabender^®^ GmbH & Co. KG, Duisburg, Germany). Filaments were pulled off by a conveyor belt (Brabender^®^ GmbH & Co. KG, Duisburg, Germany) with constant velocity set to yield filament diameters of 2.85 ± 0.1 mm and cooled with compressed air if necessary to enable complete solidification over the belt length.

#### 2.2.3. Content Uniformity (CU) of Filaments and 3D Prints

The theophylline distribution in filaments was proven by taking samples of about 100 mg every 10 cm from the filament—five of each end of every filament roll. The samples were dissolved in 0.1 N HCl and the theophylline concentrations were determined via UV/Vis spectrometry (Specocord 210plus, Analytic Jena, Jena, Germany). Polymers and additives did not influence the measurements. The content uniformity (CU) of 3D-printed full material cylinders was determined in the same way.

#### 2.2.4. He-Pycnometry

Raw material densities and densities of filaments and 3D-printed tablets were measured by helium pycnometry (UltraPyc 1200e; Quantachrome GmbH & Co. KG, Duisburg, Germany). Five consecutive volume-measurements show a standard deviation less than 0.01 cm^3^, up to ten measurements were averaged.

#### 2.2.5. Differential Scanning Calorimetry (DSC) and Thermogravimetric Analysis (TGA)

All ingredients, filaments, and 3D-printed dosage forms were analyzed via dynamic differential scanning calorimetry (DSC) to investigate possible influences of the formulation and the process on the thermal behavior of the polymeric matrix. Therefore, each sample was tested in an aluminum crucible with a perforated lid over a heating/cooling/heating profile (HPMC-based: 20 °C/200 °C/0 °C/200 °C; PEO-based: 20 °C/160 °C/0 °C/160 °C) in a DSC apparatus (DSC 1, Mettler Toledo GmbH, Switzerland) with a heating rate of 10 K/min and a nitrogen flush of 150 mL/min inside and 50 mL/min outside of the cell.

Thermal stability of theophylline and the matrix materials was investigated via thermogravimetric analysis (TGA/DSC 1 sTAR^e^; Mettler-Toledo GmbH, Greifensee, Switzerland) conducted from 30–950 °C in air with a heating rate of 10 K/min.

#### 2.2.6. Additive Manufacturing of Solid Dosage Forms

A dual filament 3D printer (Ultimaker 3, Ultimaker, Geldermalsen, The Netherlands) operated via the Cura software (Version 2.7, Ultimaker, Geldermalsen, The Netherlands) was used for the production of samples. As only dosage forms of one material were produced in this study, only one of the printer’s nozzles was used at a time. Every printable filament (iteratively determined material-specific printing parameters for reproducible, neat samples; aberrations from the printing profile of Cura for generic poly lactic acid (PLA) filament; in Table 2) was used to produce several compact samples of cylinders (8-mm diameter, 3-mm height), rings (10-mm outer diameter, 7.21-mm inner diameter, 4-mm height) and spheres (6.6-mm diameter) with the same volume of 150.8 mm^3^ but different surface to volume ratios (sphere: 0.91-mm^−1^; cylinder: 1.17-mm^−1^; ring: 1.93-mm^−1^) and (only for HPMC15) cylinders (8-mm diameter, 3-mm height) with different open porosity (20%, 50%, 80%). The mass uniformity (MU) of printed dosage forms was checked by weighing.

#### 2.2.7. Oscillatory Rheology

A rotary rheometer (MCR 302, Anton Paar GmbH, Graz, Austria) equipped with a heated parallel plate with a diameter of 25 mm was applied to determine the melt viscosity of extruded samples. After melting the sample, a gap of 1 mm was adjusted and measurements were conducted as temperature sweep with constant amplitude (0.36°) and frequency (1 Hz), both approved by amplitude and frequency sweep tests. Each sample was heated to the maximum of a material-specific temperature ramp (PEO-based: 40–120 °C; HPMC-based: 120–200 °C) then moderately cooled down while the rotor was already sweeping and afterwards reheated to the maximum temperature again. During reheating 16 data points were collected each over an integration time of 20 s and an aberration tolerance of 0.2%.

#### 2.2.8. Mechanical Testing (Three-Point Bending and Brazilian Test)

Mechanical stability of the filaments and 3D-printed tablets was tested using a material tester (Z2.5, Zwick GmbH & Co. KG, Ulm, Germany). The flexural strength of the filaments was tested via three-point-bending with a span distance of 16 mm and a speed of 10 mm/min. The tablets’ tensile strength was investigated with the Brazilian test (diametral breakage test) with a speed of 10 mm/min.

#### 2.2.9. Microstructural Investigation via Micro-Computer Tomography (µCT) and SEM

Microstructure of dosage forms was investigated via µCT imaging using a micro-computer tomographic apparatus (MicroXCT-400 Xradia; Zeiss, Oberkochen, Germany). Furthermore, the surface of different dosage forms and filaments was recorded with a scanning electron microscope (SEM) (Helios G4 CX; FEI Deutschland GmbH, Dreieich, Germany) after being sputtered with 6-nm of platinum in a vacuum coater (EM ACE 600; Leica Mikrosysteme GmbH, Wetzlar, Germany).

#### 2.2.10. Disintegration Behavior and Dissolution Testing of Dosage Forms

Disintegration of the full material cylinders was measured with an automatic apparatus (DT50, Sotax AG, Aesch, Switzerland) in 0.1 N HCl at 37 °C.

Theophylline release was investigated with an automated USP4 flow cell device (CE7 smart; Sotax AG, Aesch, Switzerland) including an UV/Vis spectrometer (Specord 200plus; Analytic Jena, Jena, Germany). All tests were conducted at 37 °C using 0.1 N HCl for the first two hours and then automatically switching to phosphate-buffered solution at pH = 6.8. The sample-specific flowrates can be obtained from Table 3.

## 3. Results and Discussion

### 3.1. Filament Characteristics

#### 3.1.1. Raw Material Particle Characteristics

**Figure 1:**
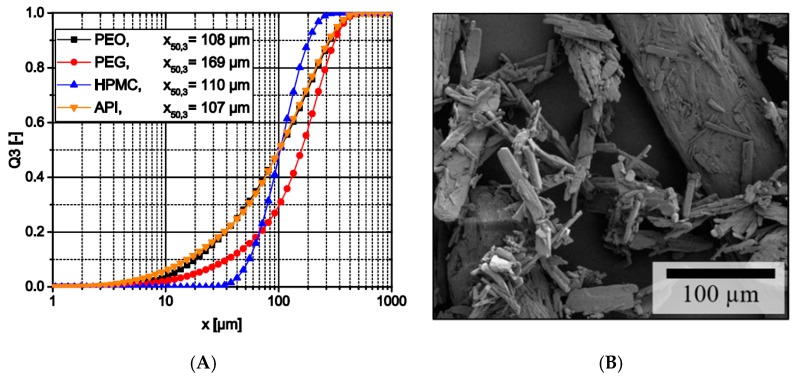
(**A**) Particle size distribution of raw materials; (**B**) SEM image of theophylline crystals.

All raw materials had relatively broad particle size distributions (Figure 1A). Except PEG, all materials had nearly the same median particle size of about 110 µm and PEO and theophylline nearly had the same size distribution. However, PEG with a median particle size of 169 µm is still in the same size range. Therefore, a segregation due to different particle sizes is unlikely. However, other properties like the materials’ true densities or the particle morphology like the acicular shape of the theophylline particles (Figure 1B) might still affect segregation of particle mixtures.

#### 3.1.2. Development of True Densities along the Process Chain

Filaments were successfully produced with a consistent diameter of 2.85 ± 0.05 mm. An increase of theophylline load resulted in a whitening of the light brown/yellowish HPMC and the yellowish/greenish PEO, depicting the increase in crystalline theophylline particles. Theoretical densities (Table 4) of all filaments were calculated using the formulation and the measured true densities of raw materials. As theophylline had a significantly higher true density, the density of the formulations increases with the theophylline load. The densities of all filaments and 3D-printed cylinders of all printable materials were determined and the deviation from the theoretical densities was calculated (Table 4). The negative difference of filament densities compared with theoretical densities allows an estimation of the closed pore volume within the filaments. As there is a trend towards higher densities along the process chain from filament to 3D print, these closed pores are opened and deaerated, respectively, during the tapering of the filament inside the 3D-printer’s hot nozzle. The significant decrease of the density of HPMC and HPMC15 during the 3D print indicates enclosed air between the single layers. To the best of the authors’ knowledge, the present work is the first to use the development of true densities along the process chain as a quality assurance tool in pharmaceutical research. However, as enclosed air, both in filaments and 3D prints, can alter the predefined properties like MU, CU, floatability, and dissolution behavior, porosity of filaments for 3D-printing of dosage forms should thoroughly be considered.

#### 3.1.3. Microstructure of Filaments

**Figure 2:**
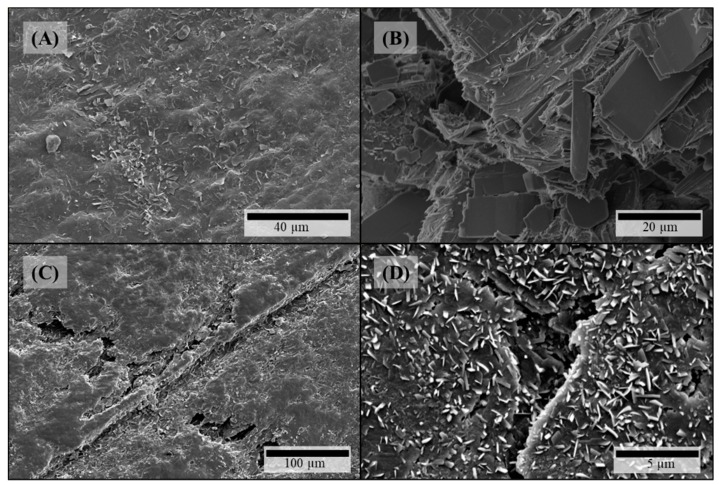
SEM pictures of HPMC-based filaments: (**A**) HPMC15 surface; (**B**) HPMC15 fracture face; (**C**) HPMC50 surface; (**D**) HPMC50 fracture face.

**Figure 3:**
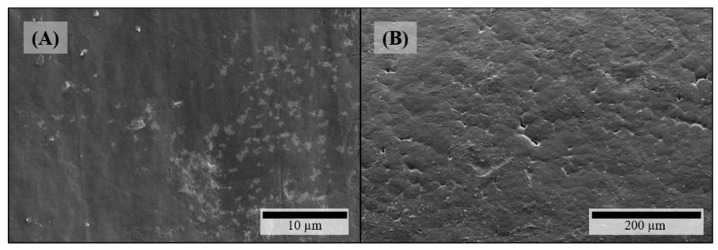
SEM pictures of filaments without any API: (**A**) HPMC surface; (**B**) PEO surface.

Microstructural investigation of HPMC-based filaments via SEM depicted all the same visible effects of theophylline particles on both surface and fracture face which got more distinct with increasing theophylline load. On the filaments’ surfaces crystalline structures occur (Figure 2A,C). As there are no comparable structures on HPMC filaments without theophylline (Figure 3A), these crystals can be identified as theophylline. In addition, defects on the crystals arise with increasing theophylline load (Figure 2C). Views of the fracture faces of theophylline-loaded HPMC filaments show the elongated theophylline crystals mainly in line with the extrusion direction of the filament (Figure 2B). In addition, some tooth-shaped structures appear on the fracture face at higher theophylline loads (Figure 2D) between the larger theophylline crystals which displays the higher amount of small theophylline particles, which are dispersed in the polymer matrix without orientation.

**Figure 4:**
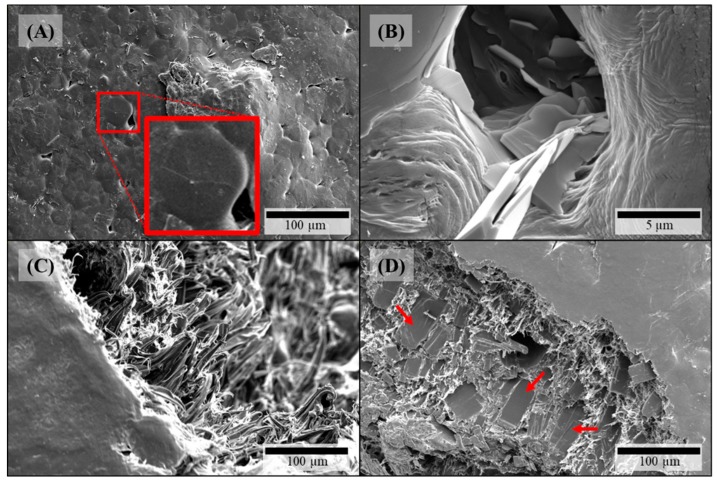
SEM pictures of PEO-based filaments: (**A**) PEO15-P surface, insert: magnification of octagonal structure; (**B**) PEO15-P surface close-up; (**C**) PEO15 fracture face; (**D**) PEO35 fracture face.

**Figure 5:**
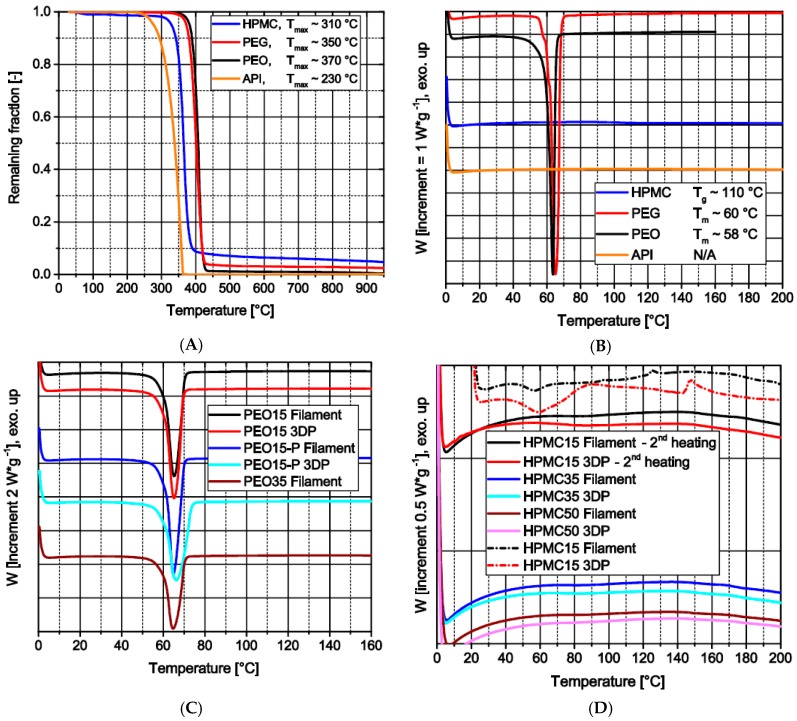
Thermal analysis: (**A**) TGA profiles of raw materials (under oxygen); (**B**) DSC profiles of raw materials (2nd heating); (**C**) DSC profiles of PEO-based materials (2nd heating); (**D**) DSC profiles of HPMC-based products (1st and 2nd heating).

On the surfaces of theophylline-loaded filaments based on PEO, theophylline crystals were also visible (Figure 4A,B), which could not be observed on the surface of pure PEO filament (Figure 3B). Furthermore, the surfaces of all PEO-based filaments exhibited octagonal structuring (Figure 4A: magnified insert) depicting the crystalline part of the semi-crystalline polymer accordingly to the crystal structure of PEO [36]. However, as the polymer PEO also consists of an amorphous part with a glass transition temperature below 0 °C (cf. Figure 5B), significant plastic yielding can be observed at fracture faces (Figure 4D). Similar to HPMC-based, theophylline-loaded filaments, PEO filaments with theophylline show elongated particles mainly in line with the extrusion direction on the inside (Figure 4C). However, small theophylline crystals without orientation between the bigger ones, as they were observed in HPMC50 filaments (Figure 2D), could not be observed within PEO-based filaments. Therefore, these small crystals may be induced by the significantly higher melt viscosity of HPMC and the associated higher shear and dispersing during extrusion.

Due to the observations during microstructural investigation of filaments, particle-related effects on the FLM process can be expected. The theophylline crystals on the filament surface could get abraded during the tapering of the diameter inside of the hot nozzle where they could accumulate and degrade due to long exposure to heat. Furthermore, the aligned elongate particles can cause temporary blockage of the hot nozzle as it has been reported before by Kempin et al. [27].

#### 3.1.4. Thermal Analysis along the Process Chain

All raw materials show thermal stability within the temperature range of all processes (Figure 5A). Theophylline is the first of the used materials to degrade at above 200 °C [35], here approximately 230 °C. The slight mass decrease of HPMC around 100 °C indicates the loss of water bound during storage, which corresponds to the USP guideline [37]. This loss of water, around 1.5 wt.%, may induce inaccuracies in the theophylline load of filaments as the water evaporates after weighing during the extrusion through the venting hole of the extruder. Furthermore, the raw materials show different thermal events within the temperature range (Figure 5B). Theophylline does not show any event up to 200 °C. However, PEG and PEO show a significant melting at around 60 °C, depicting the crystalline part of the polymers. The amorphous HPMC has a glass transition temperature around 110 °C.

Each produced filament based on PEO and each 3D print made with these filaments only show one melting event within the temperature range around 60 °C (Figure 5C). The only remarkable observation is the decrease of the specific melting enthalpy for samples with increasing theophylline load which displays the higher load of nonmelting material.

During the first heating in the course of DSC measurements of filaments and 3D prints based on HPMC, two events could be detected (Figure 5D; dashed lines): The first endothermic event at around 60 °C presumably shows the melting of the PEG contained in the formulation; the second event is exothermic and occurs from 140–160 °C. This may indicate a recrystallization of amorphous theophylline. However, as during the second heating no significant events could be detected by other means and to the best of the authors’ knowledge this behavior has not been described before, the effect is not safely attributed to these physical phenomena. In future work, this will be further investigated as an effect on printability and storage stability of filament and 3D-print.

#### 3.1.5. Theophylline Content and Distribution in Filaments

The average theophylline content of all loaded filaments is near the adjusted loads through formulation (Table 5). The slightly higher theophylline contents of the HPMC-based filaments, especially of HPMC15, may be induced by evaporation of water absorbed on the HPMC powder, as described above. Except for PEO15-P and PEO35-PL, all filaments show a deviation of theophylline content along the spool length which may be induced by an instable process or segregation of the premixed powder blends. Especially the HPMC-based filaments show an increasing content deviation along the spool length with increasing matrix fraction. As described above, this may be a result of the significantly higher particle size of PEG.

The homogeneous theophylline distribution along the whole filament spool is crucial as one spool is several meters long but the amount which is needed for one tablet is only several millimeters of filament. Therefore, a thorough understanding of the production process of a filament is necessary to produce dosage forms with uniform theophylline content.

#### 3.1.6. Influence of Polymer Matrix and Theophylline Load on Flexural Strength of Filaments

**Figure 6:**
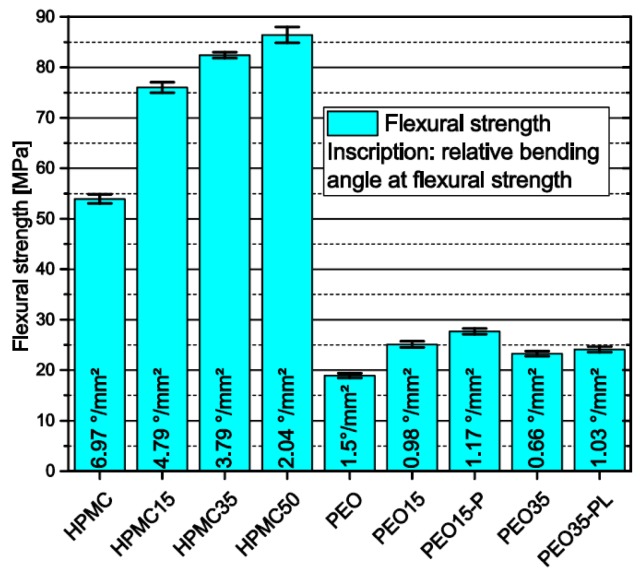
Flexural strength and relative bending angle of filaments measured via three-point-bending span = 16 mm; *n* = 6.

As intense stresses are applied to filaments during FLM, they need good mechanical stability. The flexural strength measured via three-point-bending combined with the relative flexural angle—which was derived by the bending angle of the filaments at flexural strength divided by the cross-sectional area—were taken as surrogates for the processability of filaments in FLM. In general, the results show that filaments made of HPMC have higher flexural strength than those made of PEO (Figure 6). Furthermore, an increase in flexural strength and a decrease in the relative bending angle were observed for HPMC-based filaments with increasing theophylline load. This shows that the needle-like theophylline crystals stiffen the HPMC matrix and shift the material behavior from elastic to more rigid and brittle. Filaments based on PEO first show an increase in flexural strength and decrease in bending angle as observed for HPMC. However, increasing the theophylline load from 15 wt.% to 35 wt.% weakens the filaments as a decrease in the flexural strength occurs. In both filaments an addition of PEG or PEO with different molecular weight leads to a slight increase in both flexural strength and relative bending angle.

Zhang et al. reported that filaments should exhibit a minimum flexural strength of 28.85 MPa and a maximum bending angle of 9.15° to be suitable for FLM [28]. As they do not report the filament diameters, the relative flexural angle cannot be calculated and therefore not compared with our results. However, as the flexural strength should be independent of the filament diameter the minimum flexural strength reported should be adaptable to our results. The only two filaments that were not suitable for FLM due to extensive breakage during the forwarding of the filament inside the 3D-printer were PEO35 and PEO35-PL. This shows that the value reported by Zhang et al., which is significantly higher than the flexural strength of the other PEO filaments cannot be applied as a universal rule for FLM filaments. Other characteristics like the tensile strength as reported before [31,38], or the abrasion resistance or the FLM machinery properties should also be considered to identify the suitability of filaments for FLM and will be analyzed in future work.

#### 3.1.7. Influence of Theophylline Load on Melt Viscosity of Prepared Filament Formulations

**Figure 7:**
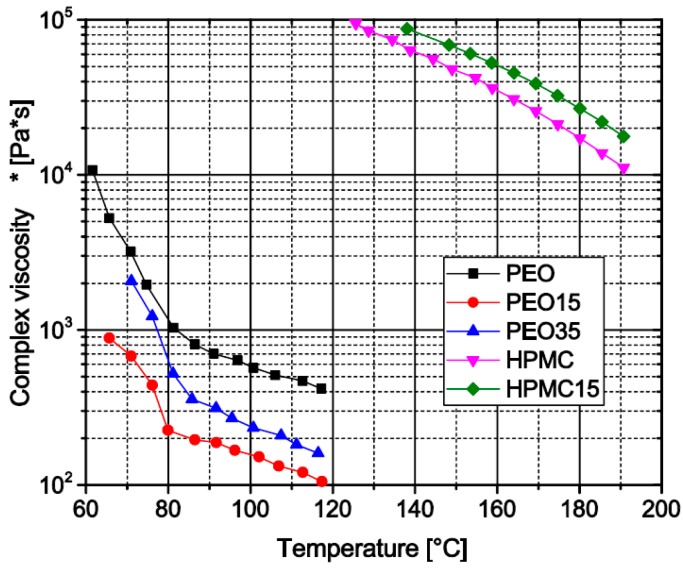
Oscillatory melt viscosity of different filament formulations (heating graph).

In both the twin-screw extrusion of filaments and FLM, the melt viscosity of the applied formulation is a crucial parameter. To determine the influence of different theophylline loads on the melt viscosity of the used matrix polymers, the complex viscosity was determined via oscillatory viscosimetry at constant amplitude and frequency during the reheating of the sample in the temperature region of the extrusion and FLM processes. The measurements show that pure PEO has a higher melt viscosity than the formulations containing theophylline, which indicates a plasticizing effect (Figure 7). However, with increasing theophylline load from 15 wt.% towards 35 wt.% the melt viscosity again increases. Compared to the data of Van Renterghem et al. [21] the initial viscosity decrease may emanate from partly dissolved theophylline in the polymer matrix while the increasing amount of particulate theophylline at higher loads again increases the melt viscosity. The high temperatures needed to prepare HPMC-based samples for viscosimetry slowly induce degradation of the material visible by a brownish discoloration. Only the pure HPMC matrix and the sample with 15 wt.% theophylline melted sufficiently for rheological analysis before significant degredation. The measured viscosity curves (Figure 7) show higher viscosities for the theophylline -loaded material. Therefore, the plastifying effect of the dissolved theophylline, if there is any, cannot outweigh the thickening effect of non-dissolved theophylline particles.

### 3.2. Characteristics of 3D-Printed Dosage Forms

#### 3.2.1. Feasibility of the Additive Production of Dosage Forms and their Mass and Content Uniformity

**Figure 8:**
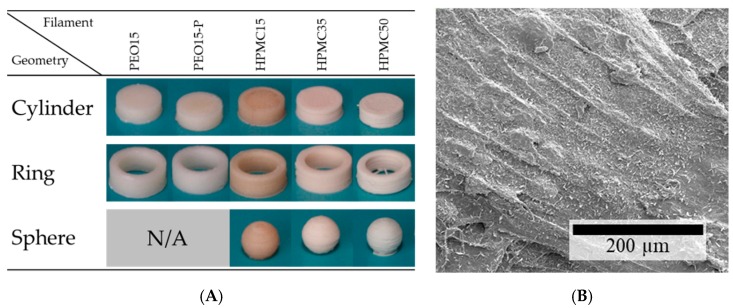
(**A**) Images of 3D-printed theophylline-loaded dosage forms with the same volume; (**B**) SEM image of the surface of a 3D-printed object made from HPMC15 filament.

Except for PEO35 and PEO35-PL, all filaments were successfully used in an FLM machine to produce different geometries with the same volume (Figure 8A). The formulation-specific printing parameters were established iteratively (data not shown). It could be determined that the nozzle temperature is the most important parameter with a narrow range; at too low temperatures the material has a too high viscosity, which can lead to a too-low extrusion and to damage of the filament in the feed unit; at too high temperatures the printed lines smear and the printed structures have an inhomogeneous surface and patterns cannot be reproducibly manufactured. During the production process of PEO-based materials, a thorough cooling was needed to gain neat structures due to the relatively low melt viscosity of PEO compared to the melting point of the crystalline fraction. Therefore, no complex structures like spheres could be produced from PEO-based filaments. FLM with HPMC-based filaments was hardly challenging. Only the temperature of the printhead nozzle and the build plate had to be raised with increasing theophylline load to gain smooth layers with good adhesion. This may be attributed to the higher amount of non-melting material as more particle-particle contacts occur with weaker interactions than the polymer parts that melt into each other. However, surprisingly, the HPMC filament without any theophylline needed the highest nozzle temperatures for good layer adhesion although the melt viscosity was lower than with theophylline load. This may be explained by the missing glass transition for HPMC containing theophylline, hinting at a plasticizing effect of dissolved theophylline molecules in the HPMC matrix. The higher viscosity induces lower interfacial forces between the printed layers as layers cannot be attached to each other smoothly. Another possible reason for the weaker layer adhesion could be the rougher surface of theophylline-loaded HPMC formulations as it can be observed in the SEM pictures (cf. Figure 2C, Figure 3A and Figure 8B). The amount of small visible theophylline crystals on the surface of 3D-printed objects is much higher than on filaments. This may indicate a post-process recrystallization on the 3D-print surface, which experienced high temperatures through direct nozzle contact.

**Figure 9:**
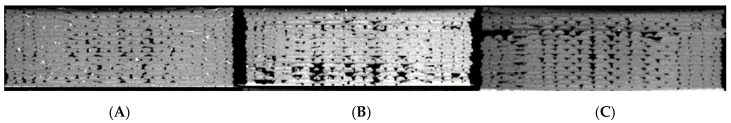
Microstructural cross section of 3D-printed HPMC cylinders via µCT (**A**) HPMC15, (**B**) HPMC35, (**C**) HPMC50.

Although every formulation based on HPMC was suitable for FLM, the accuracy decreased with increasing theophylline loads. The microstructural defects which were detected via µCT (Figure 9) were presumably, as discussed above, induced by temporary nozzle blockage which has already been reported elsewhere [27]. These structural defects can cause deviations in mass and content uniformity if the blockages are not detected during the process. Therefore, intensive studies are necessary for a better understanding of the relationship between the process, the formulation and the final properties, especially at high API loads.

With increasing theophylline loads for both HPMC and PEO-based printed cylinders and spheres, the adverse variances from the theoretical masses also increased (Table 6). The fact that some formulations show better results for the printed rings, for example HPMC15, is induced by printing parameters as the rings did not have an “infill” structure, which are printed with slightly different settings like faster print speed and acceleration. Furthermore, the theophylline content in the cylinders based on HPMC and PEO is near to the expected values. However, it has to be taken into account that the theoretical values are based on the measured weight. Therefore, the same effect of increasing adverse variance with increasing theophylline load could be detected by content determination.

#### 3.2.2. Influence of Formulation and Production on the Tensile Strength of 3D-Printed Cylinders

**Figure 10:**
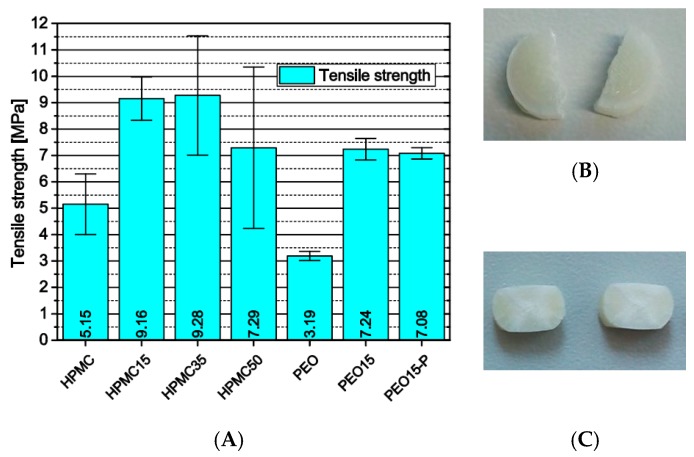
(**A**) Tensile strength of 3D-printed cylinders via Brazilian test, *n* = 6; (**B**) PEO cylinders after test; (**C**) PEO15 cylinders after test.

Another influence of the previously described process-related microstructural defects in HPMC-based samples emerged from the investigation of the mechanical properties of printed full-material cylinders via Brazilian test. Instead of a material property, the diametric compression test yielded a value more dependent on layer adhesion. Neglecting the total values (Figure 10A), the significant increase in standard deviation with increasing theophylline load represents on one hand the increasing number of defects and their statistical occurrence. On the other hand, the matrix volume decreases with increasing theophylline load inducing more particle-particle contacts with weaker interfacial attraction. The significantly lower tensile strength of HPMC cylinders without theophylline confirms the above-discussed hypothesis that the theophylline particles enhance the layer adhesion and mechanical filament properties in some way.

The PEO-based cylinders did not delaminate under diametric pressure. Instead, the pure PEO cylinders depicted nearly perfect diametric breakage as it can be observed with standard compressed tablets (Figure 10B). The cylinders containing theophylline, however, did not break but were compressed (Figure 10C). The difference is induced by dissolved theophylline molecules inside the PEO matrix plastifying the polymer and by the needle-like shape of the theophylline particles. The elongate particles are pulled out of the matrix during material failure and therefore induce a better adhesion between the fragments.

#### 3.2.3. Influence of Formulation and Geometry on Dissolution Profiles and Release Kinetics

**Figure 11:**
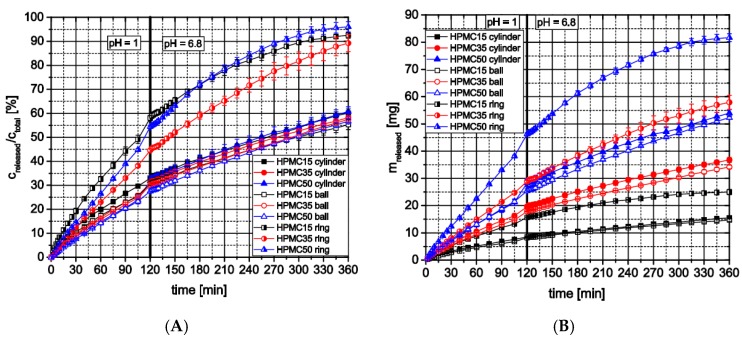
In vitro theophylline release from HPMC-based 3D-printed dosage forms using flow through apparatus; (**A**) relative release; (**B**) total release; *n* = 3.

Dissolution testing of the 3D-printed cylindrical tablets and spheres made from HPMC formulations with different theophylline loads indicated that the theophylline load had no significant influence on the release profiles (Figure 11A). All cylinders and rings of the three formulations released nearly 60% of their theophylline content over six hours. During the first two hours, being characteristic for the passage through the stomach, all release curves follow a first-order kinetic up to a theophylline release of 30%. After changing the testing medium with a shift in pH from 1 to 6.8, all curves follow a zero-order kinetic. However, the dissolution testing of printed rings with a higher surface-area-to-volume ratio showed a drug release of nearly 95% over 6 h for all formulations (compared with approx. 60% for cylindrical tablets and spheres) and higher differences between different drug loads (higher flowrate for 50 wt.% formulation must be considered) due to lower diffusion lengths within the ring structure. Of course, the theophylline mass actually released from dosage forms made with different formulations differs due to the different theophylline contents (Figure 11B). However, the same trend towards higher theophylline release from rings can be observed. These observations show that with the used HPMC grade as matrix polymer which swells in water forming a diffusion barrier, extended release dosage forms can be produced which’s release profiles are irrespective of the theophylline-content at low surface area to volume ratios and provide comparable relative release kinetics (spheres: 0.91 mm^−1^, cylinders: 1.17 mm^−1^, rings: 1.93 mm^−1^).

**Figure 12:**
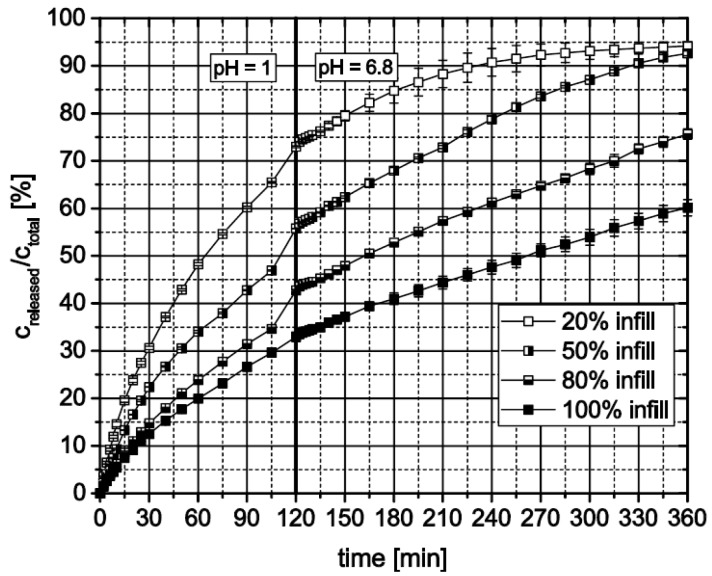
In vitro theophylline release from HPMC15 3D-printed cylinders with different open porosity defined via “infill” setting, *n* = 3.

3D-printed cylinders with open porosity controlled by the infill option of the printer’s software consist of an outer full material ring filled with a structure whose density depends on the chosen infill value [%]. Therefore, the mass of a dosage form also depends on this value. During dissolution testing of cylinders with different open porosities based on HPMC15, different behavior of the dosage forms could be observed (Figure 12). The inner structure of the dosage form with only 20% infill was washed out after several minutes leaving a ring behind. Instead of being washed out, the inner structures of both dosage forms with 50% and 80% swelled, generating a cylindrical shape. Therefore, the relatively released theophylline increases with decreasing infill. In addition, dosage forms which generate a cylinder during dissolution testing release the theophylline after comparable but faster kinetic as a full material cylinder does.

Dissolution testing of dosage forms made from PEO (data shown in Supplementary Files) showed rapid theophylline release. Depending on the geometry and open porosity, 100% theophylline release was reached after 15–60 min. Therefore, PEO-based materials are suitable for fast releasing parts of polypill applications.

## 4. Conclusions

For individualization approaches of pharmaceuticals, combining different APIs into one dosage form by 3D-printing, a high API load in the respective intermediate products is needed. This makes the thorough evaluation of highly loaded filaments inevitable. However, by increasing disperse API content, properties of filaments for 3D-printing may be altered, modulating the performance along the process chain. Therefore, particle-induced effects on printability and product properties must be elucidated to finally enable the prediction of overall process performance based on formulation and API particle properties.

In general, particles inside the polymer matrix proved to particularly alter the mechanical properties of both filaments and 3D prints. The systematic adaption of specific FLM parameters can attenuate several challenges such as layer adhesion to a certain extent. However, as the influence of size and shape of particles and their behavior during multiple heating, such as in filament extrusion and FLM itself, and their interplay with dissolved API molecules in the melt are not fully understood, further investigations are required.

Additionally, higher API content can cause instabilities in the 3D-printing process, making prediction of dosage form mass and therefore content uniformity more challenging. For tested HPMC formulations, the release profile of geometries with low surface-area-to-volume ratio is independent of the API content, facilitating an independent adjustment of the dose-and-release profile as well as the application of highly loaded polymer formulations in polypill applications.

To conclude, all experimental data demonstrate the significance of the impact of the API load over the whole process chain of 3D-printing of tablets. This knowledge must be extended further to derive models that should be taken into consideration during product development of individualized medicines.

## Figures and Tables

**Table 1:** Filament formulations and extrusion die temperatures.

Designation	Formulation in Fractions [wt.%]					Extrusion Temperatures [°C]
	HPMC	PEG	PEO	PEO-L	Theoph.	
HPMC *	95.00	5.00	-	-	-	150 *
HPMC15	81.53	4.08	-	-	14.38	160
HPMC35	62.95	3.15	-	-	33.90	160
HPMC50 **	48.78	2.44	-	-	48.78	170 **
PEO	-	-	100.00	-	-	120
PEO15	-	-	85.00	-	15.00	120
PEO15-P	-	4.08	81.53	-	14.38	120
PEO35	-	-	65.00	-	35.00	120
PEO35-PL	-	-	59.09	9.09	31.82	120

*: different extruder, **: different screw speed (200 rpm).

**Table 2:** Specific printing parameter deviating from Cura generic PLA profile for every filament.

Designation	Aberration from Standard Generic PLA Profile (Cura 2.7)
HPMC	Retraction distance: 8 mm; printing temperature: 220 °C
HPMC15	Retraction distance: 8 mm
HPMC35	Retraction distance: 8 mm; buildplate temperature: 70 °C
HPMC50	Retraction distance: 8 mm; buildplate temperature: 80 °C; printing temperature: 210 °C; print cooling: disabled
PEO/PEO15	Retraction distance: 8 mm; buildplate temperature: 55 °C; printing temperature: 110 °C; print cooling: 100% fanspeed at 1st layer; print speed: 40 mm/s
PEO15-P	
PEO35	Not printable
PEO35-PL	

**Table 3:** Sample-specific flow rates for dissolution testing in flow cell apparatus.

Flowrate [mL/min]	Samples
4	HPMC15 samples (including cylinders with open porosity) HPMC35 samples
8	HPMC50 samples
16	PEO15 samples PEO15-P samples

**Table 4:** Development of true densities along the process chain (He-pycnometer) with deviation from theoretical density of formulations.

Designation	Theoretical Density * [g/cm^3^]	Filament Density [g/cm^3^]	Density Deviation Filament [%]	3D-Print Density [g/cm^3^]	Density Deviation 3D-Print [%]
HPMC	1.255	1.192	−5.29	1.182	−6.18
HPMC15	1.289	1.268	−1.66	1.237	−4.00
HPMC35	1.335	1.300	−2.63	1.309	−1.95
HPMC50	1.371	1.342	−2.12	1.356	−1.04
PEO	1.250	1.202	−3.99	1.207	−3.56
PEO15	1.286	1.251	−2.78	1.275	−0.90
PEO15-P	1.278	1.244	−2.63	1.256	−1.71
PEO35	1.335	1.277	−4.36	N/A	N/A
PEO35-PL	1.328	1.253	−5.67	N/A	N/A

* Raw material densities [g/cm^3^]: PEO (1.25), PEO-L (1.26), PEG (1.081), HPMC (1.264), theophylline (1.492).

**Table 5:** Filament investigation for homogeneity of theophylline (in table API) distribution.

Designation	Theoretical API Content [wt.%]	API Content First End [wt.%]	API Content Second End [wt.%]	API Content Average [wt.%]
HPMC15	14.38	14.71 ± 0.28	16.54 ± 0.22	15.62 ± 0.95
HPMC35	33.90	33.09 ± 0.28	34.79 ± 0.33	33.94 ± 0.90
HPMC50	48.78	49.02 ± 0.53	49.70 ± 0.43	49.36 ± 0.59
PEO15	15.00	14.17 ± 0.17	16.03 ± 0.24	15.10 ± 0.95
PEO15-P	14.38	15.34 ± 0.15	15.76 ± 0.15	15.55 ± 0.26
PEO35 *	N/A *	N/A *	N/A *	N/A *
PEO35-PL	31.82	32.27 ± 0.42	32.76 ± 0.45	32.52 ± 0.50

*: Filament was too brittle; only small strands could be produced.

**Table 6:** Theoretical and measured dosage form masses and theophylline CU of cylinders, *n* = 3–6.

Design	m_theor._ [mg]	m_cylinder_ [mg]	m_ring_ [mg]	m_sphere_ [mg]	Cylinder Theophylline Content [mg]	
					Theoretic *	Measured
HPMC	189.18	175.37 ± 1.48	190.08 ± 0.37	183.23 ± 0.37	N/A	N/A
HPMC15	186.60	176.01 ± 1.56	183.47 ± 4.78	178.70 ± 1.18	27.89 ± 1.70	25.59 ± 0.51
HPMC35	197.44	180.14 ± 6.41	191.10 ± 0.85	174.87 ± 0.66	62.76 ± 3.72	63.53 ± 3.44
HPMC50	204.55	176.64 ± 6.50	172.33 ± 0.90	186.20 ± 1.85	88.83 ± 1.99	86.92 ± 2.61
PEO	188.50	162.97 ± 4.47	174.28 ± 1.61	N/A	N/A	N/A
PEO15	185.27	176.91 ± 1.46	188.37 ± 1.77	N/A	26.69 ± 1.69	29.10 ± 0.10
PEO15-P	192.22	171.52 ± 1.68	182.18 ± 3.16	N/A	27.15 ± 0.92	27.55 ± 0.78

*: Derived from measured tablet weights and average theophylline content of filaments including standard deviation.

## Supplementary Materials

The following are available online at https://www.mdpi.com/1999-4923/11/4/194/s1, Figure S1: In vitro theophylline release from PEO-based 3D-printed dosage forms, *n* = 3.
