# Supplementary material for: Influence of High, Disperse API Load on Properties along the Fused-Layer Modeling Process Chain of Solid Dosage Forms
Source: Pharmaceutics. 2019 Apr 22;11(4):194. doi: 10.3390/pharmaceutics11040194 (PMC6523638; doi:10.3390/pharmaceutics11040194)
Supplement: Supplementary file 1 [file pharmaceutics-11-00194-s001.pdf]

pharmaceutics

Supplementary Materials: Influence of High, Disperse API Load on Properties along the Fused-Layer Modeling Process Chain of Solid Dosage Forms

Marius Tidau, Arno Kwade and Jan Henrik Finke

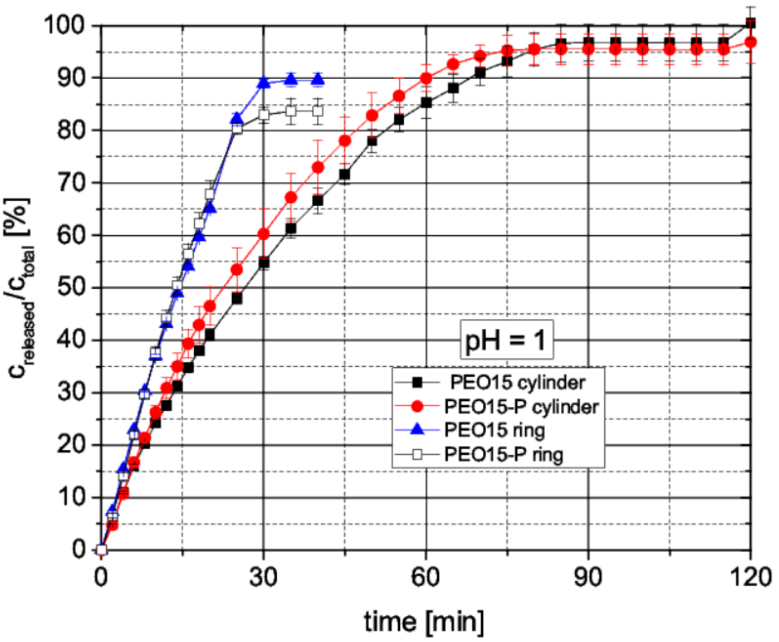

Figure S1. In vitro theophylline release from PEO-based 3D-printed dosage forms, *n* =3.
